# Impact on healthcare and operational outcomes of outsourcing to a private value-based provider: analysis of tertiary hospitals in the Community of Madrid

**DOI:** 10.3389/fpubh.2025.1652798

**Published:** 2025-09-11

**Authors:** Cristina Caramés, Javier Arcos, Bernadette Pfang, Ion Cristóbal, Juan Antonio Álvaro de la Parra

**Affiliations:** 1 Quirónsalud Healthcare Network, Grupo Hospitalario Quirónsalud, Madrid, Spain; 2 Programa de Doctorado en Medicina y Cirugía, Universidad Autónoma de Madrid, Madrid, Spain; 3 Hospital Universitario Fundación Jiménez Díaz, Madrid, Spain; 4 Clinical and Organizational Innovations Unit, Hospital Universitario Fundación Jiménez Díaz, Madrid, Spain; 5 Escuela de Doctorado UAM, Centro de Estudios de Posgrado, Universidad Autónoma de Madrid, Madrid, Spain

**Keywords:** healthcare quality, healthcare efficiency, patient safety, patient experience, outsourcing, value-based healthcare

## Abstract

**Introduction:**

Outsourcing to private healthcare providers is a topic of intense debate and with contradictory results published in the literature.

**Methods:**

In this study, we aimed to analyze the effects of outsourcing to a private provider that follows a value-based model on official indicators of quality, functioning and accessibility, safety, and patient experience recently published by the Madrid regional health system for the year 2023.

**Results:**

Notably, we found that the study hospital showed lower mortality rates, surgical and medical complications, and hospital-acquired infections than its peers. Moreover, the study hospital had significantly shorter length of stay and surgical backlog, highlighting its high levels of functioning and accessibility. Regarding patient satisfaction with the care received, the study hospital showed a significantly higher satisfaction index than the control group. Accordingly, the indicator of free choice of medical care available in the Community of Madrid showed that it was a net importer of patients from other tertiary hospitals included in this study.

**Discussion:**

Our findings support the idea that outsourcing to value-based healthcare providers represents a valid alternative that does not compromise the overall quality of healthcare offered to patients. On the contrary, this strategy could not only improve indicators but also reveal potential initiatives that will contribute to improving outcomes in public hospitals, creating a positive synergistic loop.

## 1 Introduction

As public health systems globally confront the challenges of aging populations, rising healthcare costs, and increasing patient complexity (1, 2), the question of whether outsourcing healthcare services to private organizations can improve outcomes remains deeply contested (3). The sustainability of healthcare systems is under pressure worldwide, and the privatization of healthcare delivery has become a central issue in political and policy debates. For those investing in public health, understanding the effects of privatization—especially within modern care models like value-based healthcare—is crucial for shaping equitable and effective policy (4, 5).

Despite the long-standing trend toward increased private sector involvement, there is still limited and inconsistent evidence comparing the quality of care between publicly and privately managed hospitals. While some existing evidence from high-income countries leans in favor of public management (3, 6, 7), other findings challenge this narrative (8–10). Many existing studies have methodological limitations, including the inclusion of diverse healthcare systems and inconsistent adjustment for contextual variables such as socioeconomic factors, regional healthcare models, and population needs. These factors complicate cross-study comparisons and have led to conflicting conclusions in the literature. The currently inconclusive results highlight a core public health insight: healthcare outcomes are not determined solely by the ownership model but by how systems are designed, governed, and implemented (4).

Spain’s National Health Service, organized under the Beveridge model, offers universal healthcare via 17 autonomous regional health authorities. One of the largest is Madrid’s Regional Health Service (SERMAS), which serves over 6.5 million people—13% of Spain’s population—through a comprehensive network of primary care centers and more than 25 hospitals, including eight tertiary-level institutions. Since 2010, the Madrid health system has allowed residents to choose their healthcare providers, including both publicly and privately managed hospitals. While most hospitals in the region are publicly managed, five—including the Fundación Jiménez Díaz University Hospital—are privately operated under a value-based healthcare (VBHC) model (11, 12). For public health stakeholders, the core concern is whether outsourcing within a VBHC model supports or undermines equity, access, and health system resilience. While VBHC principles align with many public health goals (13)—such as improving efficiency, outcomes, and patient experience—the interaction between private management and VBHC has yet to be fully explored in terms of its broader population health impact.

As systems around the world seek to modernize while preserving the principles of universal access, evidence is crucial to ensure that policy decisions enhance—not erode—public health outcomes. To fill this knowledge gap, the current study analyzes data from 2023 across eight tertiary hospitals in the Madrid region. The analysis focuses on key indicators of care quality, patient safety, functioning and accessibility, and patient satisfaction to assess how a privately managed hospital performs relative to publicly managed institutions in a value-based care context. For those in public health, this research offers important insights into how management models intersect with care quality and equity.

## 2 Methods

### 2.1 Study design and participants

A retrospective observational analysis was performed featuring data from the annual report of the Madrid health system for the year 2023. All eight tertiary hospitals from the Madrid health system were included in the analysis. The outsourced hospital was considered the study hospital, while the remaining seven constituted the control group. Data were extracted from the public available report. Secondary and primary tier hospitals, as well as pediatric, psychiatric, and long-term care facilities were excluded from the analysis.

### 2.2 Outcomes and measurements

The study assessed a number of healthcare quality indicators assessed as part of yearly quality audits performed by the Madrid health system, including quality of care and patient safety metrics [standardized hospital mortality ratio (SHMR), inpatient surgical and medical complications, and hospital-acquired infection rates], accessibility (mean surgical backlog) and functioning metrics [average length of stay, case-mix adjusted average length of stay (CMAILS)], and, and indicators of patient satisfaction (results of patient satisfaction surveys, and number of patients choosing to transfer to or away from the study hospital and control group).

### 2.3 Statistics

A descriptive analysis of the dataset was performed in which categorical variables were presented as number (percentage) and continuous variables as mean (standard deviation). Our analysis incorporated two adjusted indicators: the SHMR and CMAILS. These indicators enabled comparisons between hospitals and the standardized reference values established by the Madrid Health Service, which are set at 1. For each result, a 95% confidence interval was calculated by the Madrid Department of Health using Byar’s approximation of the exact Poisson distribution and was reported in the annual audit data. Mortality rates or expected lengths of stay lower than average were identified when the entire confidence interval was below 1, whereas values exceeding 1 indicated higher-than-average mortality or length of stay. Indicators such as the SHMR can be influenced by patient characteristics, disease severity, and health status prior to hospital admission, so risk adjustment systems have been developed for their evaluation. The methodology used in risk adjustment models for indicators such as the SHMR is used internationally and nationally (Quality Indicator Empirical Methods, v2021. Agency for Healthcare Research and Quality. https://qualityindicators.ahrq.gov/). It uses a logistic regression statistical model that controls for potential confounding factors, such as patient characteristics, to assess the effectiveness of healthcare on the indicator studied. The CMAILS is calculated using an indirect rate adjustment, comparing the length of stay that all acute care hospitals used to treat their patients during a year with the length of stay that all acute care hospitals in their group would have used during that same year. To assess differences in the prevalence of complications and infections between the study hospital and those in the control group (both individually and collectively), logistic regression analysis was conducted. Results were expressed as odds ratios (ORs), with corresponding 95% confidence intervals (CIs) and *p*-values. Additionally, Student’s t-tests were used to compare average case mix complexity, average hospital stay duration, surgical backlog, and the number of patients opting to transfer to or from their default hospital within the designated catchment area. Statistical significance was defined as a two-sided *p*-value less than 0.05. All analyses were conducted using R version 4.3.1 (R: A Language and Environment for Statistical Computing, R Foundation for Statistical Computing, Vienna, Austria).

### 2.4 Ethics and reporting standards

The study complied with the standards set forth in the Declaration of Helsinki and was granted a formal ethics waiver by the Fundación Jiménez Díaz Ethics Committee. STROBE guidelines were followed when drafting the manuscript (14).

## 3 Results

### 3.1 General parameters of the hospital cohort

A total number of 8,983,462 care episodes were reported from tertiary hospitals belonging to the Madrid Health Service during the year 2023. Of note, the study hospital recorded 1,376,626 care episodes in that year, corresponding to 15.32% of the total. To further analyze the available information, we stratified care episodes in the following subgroups: outpatient care, emergency department care, inpatient care, and surgical procedures. Data from each tertiary hospital included in the analysis are shown in Supplementary Table S1.

We observed a similar case mix complexity between the study hospital and the average complexity of the control group (1.27 vs. 1.27 ± 0.06). Data for the specific case mix complexity of each hospital are included in Supplementary Table S2.

### 3.2 Analysis of quality of care and patient safety indicators

The SHMR is an indicator of excellence to measure the quality of care, and we aimed to analyze the potential changes due to a value-based model of healthcare privatization. Notably, we found that the study hospital showed, by far, the lowest two-year SHMR of the eight tertiary hospitals included in this study (Table 1). Specifically, the value reported by the study hospital was 0.73 and the hospitals included in the control group showed a range from 0.85 to 1.15 for this indicator.

**Table 1:** Two-year standardized hospital mortality ratio for tertiary hospitals from the Madrid (Spain) Health Service during the year 2023.

Hospital	2-SHMR (95% CI)
Study hospital	0.73 (0.67–0.79)
Control 1	1.11 (1.03–1.21)
Control 2	0.85 (0.78–0.91)
Control 3	1.04 (0.97–1.11)
Control 4	0.97 (0.89–1.05)
Control 5	1.11 (1.01–1.22)
Control 6	1.06 (0.98–1.15)
Control 7	1.15 (1.06–1.24)

SHMR, Two-year Standard Hospital Mortality Ratio; CI, confidence interval.

**Figure 1:**
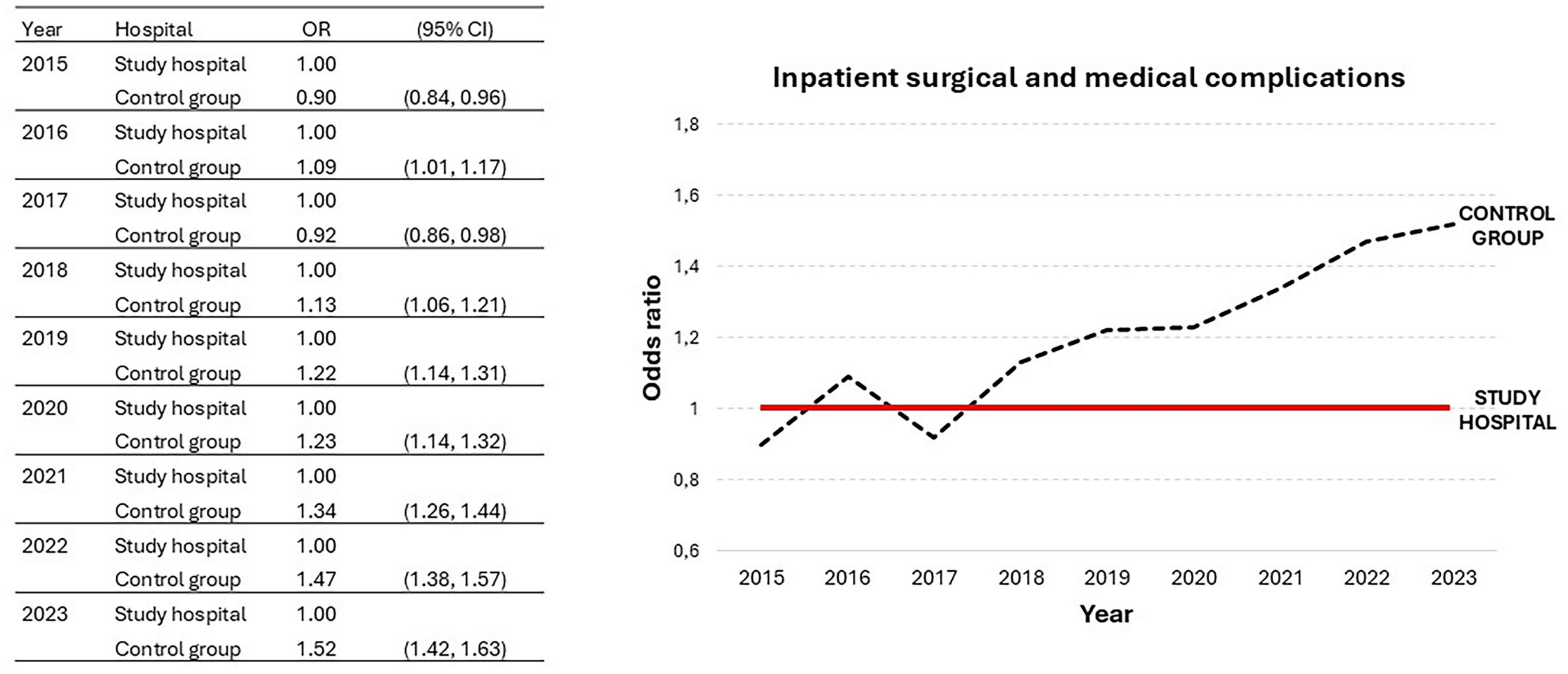
Trend in reported rates of inpatient surgical and medical complications for the study hospital and control group from 2015 to 2023; OR, odds ratio; CI, confidence interval.

Regarding patient safety indicators, we observed that the study hospital had a significantly lower probability of inpatient surgical and medical complications during the year 2023 in comparison to each of the seven hospitals included in the control group (Table 2). We also compared the study hospital to the average of the control group and, as expected, differences were markedly significant (Table 2). To achieve a more global perspective, we analyzed the trend of inpatient complications over the last decade (Figure 1). Interestingly, we observed that the study hospital shows a clear trend toward improved patient safety, with a progressively lower complication rate regarding the control group over the years.

**Table 2:** Comparisons of inpatient surgical and medical complications rates reported for the year 2023.

Hospital	OR	(95% CI)	*P*
Study hospital	1.00		
Control 1	1.74	(1.61–1.89)	
Control 2	1.50	(1.39–1.62)	
Control 3	1.54	(1.42–1.66)	
Control 4	1.38	(1.28–1.49)	
Control 5	1.65	(1.50–1.82)	
Control 6	1.64	(1.51–1.78)	
Control 7	1.39	(1.28–1.51)	
Study hospital	1.00		
Control group	1.52	(1.42, 1.63)	<0.001

OR, odds ratio; CI, confidence interval.

**Figure 2:**
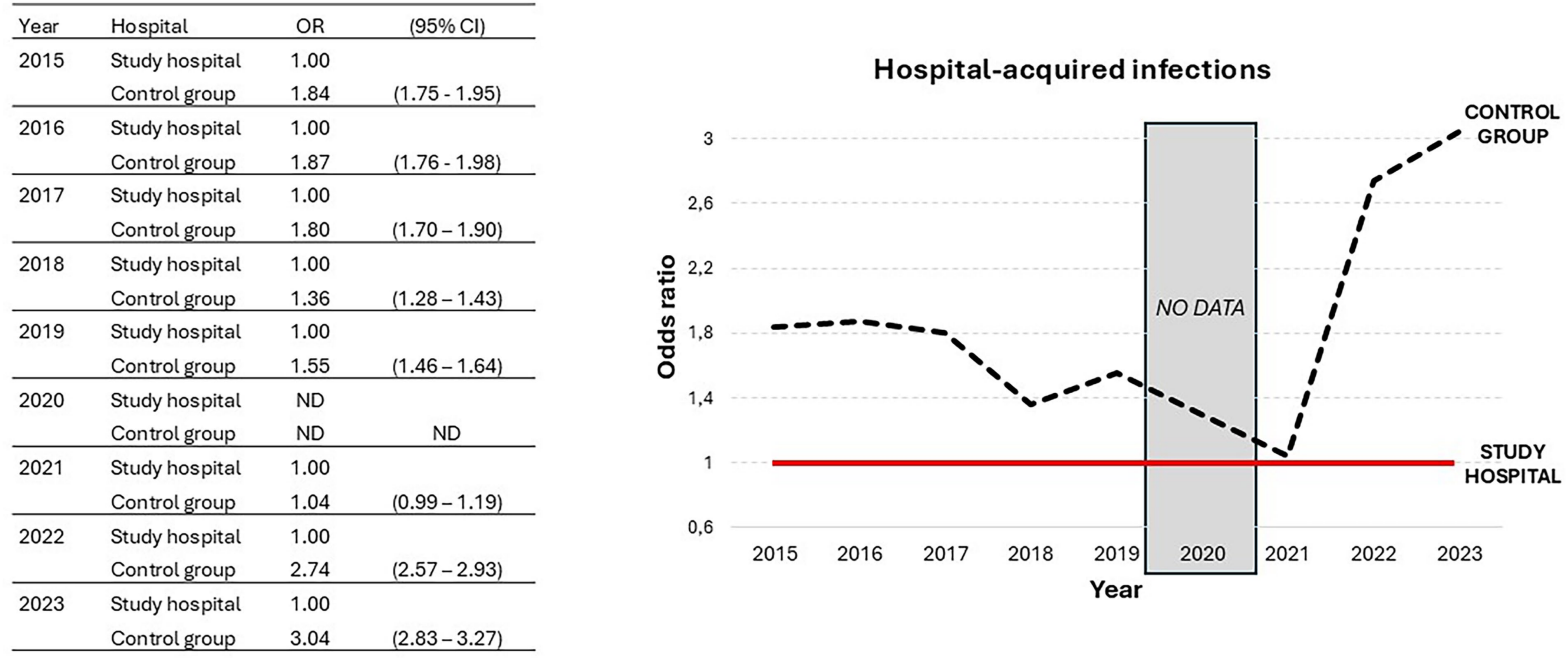
Trend in hospital-acquired infections reported for the study hospital and control group from 2015 to 2023; OR, odds ratio; CI, confidence interval.

Furthermore, we also explored hospital-acquired infection rates as an additional indicator of patient safety. In 2023, the study hospital showed the best results among the eight tertiary hospitals, with a significantly lower rate of hospital-acquired infections than the control group (OR = 3.04; CI95% = 2.83 to 3.27; *p* < 0.001) (Table 3). Moreover, this observation validates the improvement reported in 2022, thus recovering the trend toward improved patient safety observed until 2019 (data were not reported in 2020 due to the COVID-19 pandemic) (Figure 2).

**Table 3:** Hospital-acquired infection prevalence for tertiary hospitals from the Madrid Health Service reported for the year 2023.

Hospital	OR	(95% CI)	*P*
Study hospital	1.00		
Control 1	3.21	(2.96–3.48)	
Control 2	2.95	(2.73–3.19)	
Control 3	2.21	(2.04–2.40)	
Control 4	3.49	(3.24–3.77)	
Control 5	4.24	(3.89–4.63)	
Control 6	3.10	(2.86–3.37)	
Control 7	2.90	(2.68–3.15)	
Study hospital	1.00		
Control group	3.04	(2.83–3.27)	<0.001

OR, odds ratio; CI, confidence interval.

### 3.3 Potential impact in functioning and accessibility indicators

To evaluate the effects of value-based outsourcing on functioning and accessibility, we analyzed several indicators such as average inpatient length of stay, CMAILS, and mean surgical backlog. Similar to the 2021 and 2022 results, the study hospital again demonstrated the lowest average length of stay (4.63 days) among the tertiary hospitals from the Madrid health service (Supplementary Table S3). Accordingly, the study hospital showed significantly shorter-than-predicted CMAILS during 2023, with better results than the rest of the tertiary hospitals (Supplementary Table S4).

Furthermore, we observed that the study hospital had a significantly shorter mean surgical backlog than the average surgical backlog among the tertiary hospitals from the Madrid Health Service in the year 2023 (24.77 vs. 55.08 days; *p* < 0.001). In addition, we analyzed this indicator in the period pre- and post-COVID-19 and in the whole period from 2015 to 2023, aiming to identify potential deviations caused by the pandemic. Notably, we found significant differences with all the seven hospitals from the control group, and the average surgical backlog of the control group was more than three times higher than that of the study hospital (Table 4). Moreover, similar results were observed after stratifying the time series in pre- (2015–2019) (Supplementary Table S5) and post-COVID-19 (2020–2023) (Supplementary Table S6), which indicates higher accessibility levels in the study hospital.

**Table 4:** Differences in surgical backlog average (in days) between the study hospital and each of the tertiary hospitals of the control group in the period from 2015 to 2023.

Hospital	2015–2023		Control vs. Study hospital	
	Mean (SD)	Median (IQR)	Difference (95% CI)	*P*
Study hospital	15.9 (5.6)	13.3 (13.0–14.9)		
Control 1	64.6 (18.7)	55.7 (51.6–81.8)	48.7 (34.1–63.3)	<0.001
Control 2	71.9 (12.5)	67.8 (65.1–77.4)	55.9 (45.9–66.0)	<0.001
Control 3	80.0 (19.4)	78.3 (72.3–83.9)	64.1 (48.9–79.3)	<0.001
Control 4	51.6 (7.8)	54.0 (47.9–56.6)	35.7 (28.9–42.5)	<0.001
Control 5	60.5 (21.3)	59.7 (54.1–64.7)	44.5 (27.9–61.1)	<0.001
Control 6	67.1 (18.7)	65.8 (63.7–70.1)	51.2 (36.5–65.8)	<0.001
Control 7	77.7 (16.2)	74.8 (69.5–80.1)	61.8 (49.0–74.5)	<0.001
Study hospital	15.9 (5.6)	13.3 (13.0–14.9)		
Control group	67.6 (18.6)	65.5 (55.7–77.9)	51.7 (45.6–57.8)	<0.001

SD, standard deviation; IQR, interquartile range; CI, confidence interval.

### 3.4 Indicators of patient satisfaction

Overall satisfaction with the care received is a key indicator of the patient experience, providing essential information about their expectations and perceptions of the healthcare process and its quality. Results of patient satisfaction surveys for tertiary hospitals from the Madrid (Spain) Health Service are shown in Table 5. Patient satisfaction survey campaigns were not performed during 2020 due to the COVID-19 pandemic. The study hospital continued the positive trend of the previous 2 years and in 2023 again led the satisfaction index among tertiary hospitals, with more than 3 points above the control group average. Analyzing the historical series from 2015 to 2023, we see that the study hospital shows a significantly better satisfaction index than the control group.

**Table 5:** Results of patient satisfaction surveys for tertiary hospitals from the Madrid (Spain) Health Service, 2015–2023.

	2021	2022	2023	2015–2023	SD	*P*
Study hospital	91.13	92.39	92.71	92.71	0.85	
Control 1	91.3	91.77	90.52	89.24	2.56	0.004
Control 2	88.34	91.29	90.20	89.21	1.25	<0.001
Control 3	89.89	87.02	88.99	87.54	1.72	<0.001
Control 4	88.29	88.02	87.19	88.64	1.14	<0.001
Control 5	90	90.05	89.62	89.33	1.00	<0.001
Control 6	90.81	92.2	89.61	91.22	1.67	0.001
Control 7	90.14	87.87	90.37	88.52	2.16	<0.001
Study hospital	91.13	92.39	92.71	92.71	0.85	
Control group	89.82	89.75	89.50	89.10	0.90	<0.001

SD, Standard deviation.

Finally, we studied the number of patients from each hospital who chose to transfer to other centers, as well as the number of patients who were admitted from other hospitals. We measured this indicator of free choice of medical care using the ratio of admitted and discharged patients for each hospital included in this work. Of importance, this coefficient of inward transfers/outward transfers was 11.40 for the study hospital in contrast with a coefficient of 0.39 for the control group during the year 2023. This data indicates that the study hospital is a net importer of patients from the rest of tertiary hospitals except in one case in which this ratio was 1.26 (Table 6). To compare this observation with the past years we calculated that this ratio was 10 in the study hospital from 2015 to 2023 whereas it shows a value of 0.62 for the control group, highlighting that the data of 2023 reinforced the sustained trend shown over the past years (data not shown).

**Table 6:** Number of patients choosing to transfer to and from their corresponding hospital as per catchment area in the year 2023.

Hospital	2023		IT/OT
	Inward transfers	Outward transfers	
Study hospital	84,082	7,374	11.40
Control 1	13,525	22,026	0.61
Control 2	11,720	9,329	1.26
Control 3	7,121	24,274	0.29
Control 4	8,857	48,852	0.18
Control 5	3,139	7,678	0.41
Control 6	14,088	21,487	0.66
Control 7	6,897	31,789	0.22
Control group	65,347	165,795	0.39

## 4 Discussion

This work analyzes recently reported annual official public indicators from the Madrid Health Service for the year 2023. The study includes eight tertiary hospitals located in the Madrid region and aimed to evaluate the results of a public hospital outsourced to a private provider following a value-based strategy (study hospital). The comparative analysis of healthcare indicators does not support the hypothesis that outsourcing is associated with worse healthcare indicators. On the contrary, our findings highlight that, at least in this case, there is a substantial improvement in most healthcare indicators when compared to publicly managed hospitals. These findings validate other previous publications demonstrating the benefits of the value-based model and encourages the progressive migration of the rest of hospitals to this model, which improves patient outcomes.

In the year 2023 the study hospital reported 1,376,626 care episodes, representing a 15.32% of the total number of care episodes from tertiary hospitals, and a similar case-mix complexity compared to the control group. These observations are concordant with an average value of 15.53% of total care episodes recorded from the study hospital together with a similar case mix-complexity, both registered for the period from 2015 to 2022 (extracted from past published official annual data).

Of note, the study hospital showed a significant lower hospital mortality ratio compared with each of the rest tertiary hospitals (Table 1), consolidating the data reported in 2022, when it had the second-best score for this indicator (data not shown). These findings are in contrast with literature in which outsourcing was associated with increased mortality rates (7), but in accordance with another Spanish study in which mortality rates after coronary surgery were lower in outsourced hospitals (10). Regarding patient safety, our study validates the positive trend observed for the study hospital in surgical and medical complications in the past decade (Figure 1), placing it at the top of the tertiary hospitals in Madrid in the year 2023 (Table 2). This finding is similar to that of a previous study in which independent management was associated with lower case-mix adjusted complications for hip replacement, cataract surgery and hernia repair (15). Of note, the Figure 2 shows that the study hospital had a lower rate of hospital-acquired infections than the control group in all years except 2021, when no differences were observed. This finding could be influenced by the COVID pandemic, and a very marked improvement was seen in 2022. In this sense, the 2023 data validate the very significant improvement seen in this indicator in 2022. This finding is in contrast with another study in which outsourcing cleaning facilities was associated with higher hospital acquired infections (16).

We also analyzed potential differences in functioning and accessibility indicators average inpatient length of stay, CMAILS, and mean surgical backlog. Although a progressive reduction in average inpatient length of stay can be observed between 2021 and 2023 in all tertiary hospitals, the study hospital stably maintains the best data with respect to this indicator (Supplementary Table S3). Additionally, the study hospital showed significantly shorter CMAILS than expected during 2023, with much better results than the control group. These findings are in line with previous studies reporting improved efficiency indicators after increased outsourcing, including higher accessibility to care and lower healthcare-related expenditure (17–19).

Regarding patient care indicators, overall satisfaction with the care received allows us to assess the quality of care provided, identify potential areas for improvement, and align best practices with patient priorities and preferences. This indicator shows the percentage of patients satisfied with the care received at the hospital, in the areas of inpatient care, outpatient consultations, ambulatory surgery, and emergency services. In a value-based model where the patient is at the center of care, these indicators demonstrate that the model works and, notably, the study hospital had the best scores (Table 5). Furthermore, the free-choice results confirm the overall satisfaction results and highlight that this type of strategy has clear positive effects not only on clinical outcome indicators but also on patient perceptions. Thus, the free-choice results were overwhelmingly in favor of the study hospital, with an incoming patient flow 10 times greater than the outgoing patient flow in the analyzed series (Table 6). Our results are concordant with findings from another study demonstrating higher patient satisfaction with privatized care (9).

Overall, the results observed in 2023 are in line with those observed in previous years, increasing in some cases the positive trend shown by the study hospital as well as the differences with respect to the control group. However, an important limitation of our study is that being retrospective it cannot confirm the causality between the results and the value-based model. In addition, our results are context-specific and may not be applicable to different health systems in other countries or those lacking similar digital or policy frameworks. Another relevant limitation is that our study includes only one study hospital, which makes independent validation of the results necessary. Finally, we did not adjust our findings for variables such as patient socioeconomic status, infrastructure or healthcare staffing that could represent potential confounding factors.

## 5 Conclusion

The findings described in our study validate the study hospital’s commitment to the value-based strategy, which has been deepened with the progressive implementation of numerous initiatives at the study hospital, and which have made this hospital a reference center for VBHC in Spain (11, 12, 20–24). The results of these initiatives have been driving their adoption in other public hospitals, which demonstrates how outsourcing to private health providers that follow a value-based model not only offers and demonstrates better results but also serves to improve the entire public health system, which is adopting these initiatives due to their association with better results for patients, as well as the quality and functioning and accessibility of the healthcare delivered. In summary, the value-based model represents a very important change with respect to the traditional way of providing healthcare, since it is a model based on processes and not on acts, which promotes proactive medicine rather than reactive medicine, and which places the patient at the center of the healthcare process, in which they become a proactive protagonist. Healthcare policy makers should consider outsourcing to value-based providers as a sustainable strategy for health systems worldwide.

## Data Availability

The original contributions presented in the study are included in the article/Supplementary material, further inquiries can be directed to the corresponding authors.
