# Correction: Impact on healthcare and operational outcomes of outsourcing to a private value-based provider: analysis of tertiary hospitals in the Community of Madrid

**DOI:** 10.3389/fpubh.2026.1840423

**Published:** 2026-04-10

**Authors:** Cristina Caramés, Javier Arcos, Bernadette Pfang, Ion Cristóbal, Juan Antonio Álvaro de la Parra

**Affiliations:** 1 Quirónsalud Healthcare Network, Grupo Hospitalario Quirónsalud, Madrid, Spain; 2 Programa de Doctorado en Medicina y Cirugía, Universidad Autónoma de Madrid, Madrid, Spain; 3 Hospital Universitario Fundación Jiménez Díaz, Madrid, Spain; 4 Clinical and Organizational Innovations Unit, Hospital Universitario Fundación Jiménez Díaz, Madrid, Spain; 5 Escuela de Doctorado UAM, Centro de Estudios de Posgrado, Universidad Autónoma de Madrid, Madrid, Spain

**Keywords:** healthcare quality, healthcare efficiency, patient safety, patient experience, outsourcing, value-based healthcare

Affiliation “Programa de Doctorado en Medicina y Cirugía, Universidad Autónoma de Madrid, Madrid, Spain” was omitted from the paper. This affiliation has now been added for author Cristina Caramés.

Affiliation “Escuela de Doctorado UAM, Centro de Estudios de Posgrado, Universidad Autónoma de Madrid, Madrid, Spain” was omitted from the paper. This affiliation has now been added for author Juan Antonio Álvaro de la Parra.

The original version of this article has been updated.

