# Supplementary material for: Impact on healthcare and operational outcomes of outsourcing to a private value-based provider: analysis of tertiary hospitals in the Community of Madrid
Source: Front Public Health. 2025 Sep 11;13:1652798. doi: 10.3389/fpubh.2025.1652798 (PMC12460369; doi:10.3389/fpubh.2025.1652798)
Supplement: Supplementary file 1 [file Table_1.doc]

**Table S1.** Episodes of outpatient care, emergency department care, inpatient care, and surgical procedures from tertiary hospitals belonging to the Madrid health service during the year 2023.

|  | Outpatient care | Emergency care | Inpatient care | Surgical procedures |
| --- | --- | --- | --- | --- |
| Study Hospital | 1,113,617 | 194,452 | 29,901 | 38,656 |
| Control 1 | 788,010 | 149,779 | 29,980 | 29,084 |
| Control 2 | 852,984 | 272,287 | 43,595 | 31,802 |
| Control 3 | 962,162 | 309,665 | 44,764 | 37,548 |
| Control 4 | 1,013,411 | 252,397 | 48,637 | 35,589 |
| Control 5 | 458,601 | 114,636 | 15,337 | 14,708 |
| Control 6 | 596,890 | 195,817 | 27,365 | 20,772 |
| Control 7 | 1,032,643 | 165,027 | 32,895 | 30,451 |
| Total | 6,818,318 | 1,654,060 | 272,474 | 238,610 |
