# Supplementary material for: Impact on healthcare and operational outcomes of outsourcing to a private value-based provider: analysis of tertiary hospitals in the Community of Madrid
Source: Front Public Health. 2025 Sep 11;13:1652798. doi: 10.3389/fpubh.2025.1652798 (PMC12460369; doi:10.3389/fpubh.2025.1652798)
Supplement: Supplementary file 2 [file Table_2.doc]

**Table S2.** Average case mix complexity (excluding obstetric patients) for tertiary hospitals from the Madrid (Spain) health service during the year 2023.

| **Hospital** | **Year 2023** |
| --- | --- |
| Study Hospital | 1.27 |
| Control 1 | 1.2 |
| Control 2 | 1.36 |
| Control 3 | 1.28 |
| Control 4 | 1.29 |
| Control 5 | 1.22 |
| Control 6 | 1.32 |
| Control 7 | 1.23 |
| Average of the control group | 1.27 |
| Standard Deviation | 0.06 |
