# Supplementary material for: Impact on healthcare and operational outcomes of outsourcing to a private value-based provider: analysis of tertiary hospitals in the Community of Madrid
Source: Front Public Health. 2025 Sep 11;13:1652798. doi: 10.3389/fpubh.2025.1652798 (PMC12460369; doi:10.3389/fpubh.2025.1652798)
Supplement: Supplementary file 3 [file Table_3.docx]

|  | 2021 | 2022 | 2023 |
| --- | --- | --- | --- |
| Study hospital | 5.39 | 4.86 | 4.63 |
| Control 1 | 6.39 | 6.24 | 5.89 |
| Control 2 | 6.7 | 6.51 | 6.33 |
| Control 3 | 6.33 | 6.04 | 5.74 |
| Control 4 | 5.86 | 5.81 | 5.63 |
| Control 5 | 7.11 | 6.7 | 6.46 |
| Control 6 | 6.41 | 6.07 | 5.91 |
| Control 7 | 6.31 | 5.99 | 5.77 |

**Table S3.** Average inpatient length of stay in days for the study hospital and each of the hospitals from the control group, 2021–2023.
