# Supplementary material for: Impact on healthcare and operational outcomes of outsourcing to a private value-based provider: analysis of tertiary hospitals in the Community of Madrid
Source: Front Public Health. 2025 Sep 11;13:1652798. doi: 10.3389/fpubh.2025.1652798 (PMC12460369; doi:10.3389/fpubh.2025.1652798)
Supplement: Supplementary file 4 [file Table_4.docx]

**Table S4.** CMAILS rate for tertiary hospitals from the Madrid Health Service in 2023.

| Hospital | CMAILS | 95% CI |
| --- | --- | --- |
| Study Hospital | 0.819 | 0.814 – 0.823 |
| Control 1 | 1.063 | 1.058 – 1.069 |
| Control 2 | 1.035 | 1.030 – 1.039 |
| Control 3 | 0.999 | 0.995 – 1.003 |
| Control 4 | 1.025 | 1.021 – 1.029 |
| Control 5 | 1.056 | 1.049 – 1.063 |
| Control 6 | 1.001 | 0.996 – 1.006 |
| Control 7 | 1.008 | 1.003 – 1.013 |

CMAILS: Case-mix adjusted average inpatient length of stay; CI: confidence interval
