# Supplementary material for: Impact on healthcare and operational outcomes of outsourcing to a private value-based provider: analysis of tertiary hospitals in the Community of Madrid
Source: Front Public Health. 2025 Sep 11;13:1652798. doi: 10.3389/fpubh.2025.1652798 (PMC12460369; doi:10.3389/fpubh.2025.1652798)
Supplement: Supplementary file 5 [file Table_5.docx]

**Table S5.** Differences in surgical backlog average (in days) between the study hospital and each of the tertiary hospitals of the control group in the period from 2015 to 2019.

|  | 2015 - 2019 | |  | Control vs. Study hospital | |
| --- | --- | --- | --- | --- | --- |
| Hospital | Mean (SD) | Median (IQR) |  | Difference (95% CI) | P |
| Study hospital | 15.6 (6.2) | 13.3 (13.0 -13.4) |  |  |  |
| Control 1 | 54.3 (10.8) | 53.8 (51.6 – 55.7) |  | 38.7 (25.9 – 51.6) | <0.001 |
| Control 2 | 66.4 (7.6) | 65.5 (65.1 – 67.8) |  | 50.8 (40.7 – 60.9) | <0.001 |
| Control 3 | 78.5 (4.44) | 78.3 (76.8 - 81.3) |  | 62.9 (55.0 - 70.7) | <0.001 |
| Control 4 | 50.4 (8.8) | 54.0 (50.7 - 55.6) |  | 34.8 (23.7 – 45.9) | <0.001 |
| Control 5 | 50.6 (14.9) | 54.1 (46.5 - 59.7) |  | 35.0 (18.4 – 51.6) | 0.001 |
| Control 6 | 59.0 (16.5) | 65.7 (63.7 – 65.8) |  | 43.4 (25.3 – 61.6) | 0.001 |
| Control 7 | 75.8 (19.2) | 74.8 (69.5 – 79.6) |  | 60.2 (39.4 – 81.0) | <0.001 |
| Study hospital | 15.6 (6.2) | 13.3 (13.0 -13.4) |  |  |  |
| Control group | 62.2 (15.9) | 63.7 (54.1 – 71.3) |  | 45.6 (31.9 – 61.2) | <0.001 |

SD, Standard Deviation; IQR, Interquartile range; CI, Confidence interval.
