# Supplementary material for: Impact on healthcare and operational outcomes of outsourcing to a private value-based provider: analysis of tertiary hospitals in the Community of Madrid
Source: Front Public Health. 2025 Sep 11;13:1652798. doi: 10.3389/fpubh.2025.1652798 (PMC12460369; doi:10.3389/fpubh.2025.1652798)
Supplement: Supplementary file 6 [file Table_6.docx]

**Table S6.** Differences in surgical backlog average (in days) between the study hospital and each of the tertiary hospitals of the control group in the period from 2020 to 2023.

|  | 2020 - 2023 | |  | Control vs. Study hospital | |
| --- | --- | --- | --- | --- | --- |
| Hospital | Mean (SD) | Median (IQR) |  | Difference (95% CI) | P |
| Study hospital | 16.3 (5.8) | 14.1 (13.0 - 17.4) |  |  |  |
| Control 1 | 77.5 (19.5) | 84.1 (73.6 - 88.0) |  | 61.2 (36.3 - 86.0) | 0.001 |
| Control 2 | 78.6 (15.2) | 80.1 (71.2 - 87.5) |  | 62.3 (42.5 - 82.2) | <0.001 |
| Control 3 | 82.0 (31.2) | 76.6 (61.5 - 97.1) |  | 65.7 (16.9 - 114) | 0.0023 |
| Control 4 | 53.1 (7.21) | 52.9 (47.4 - 58.6) |  | 36.8 (25.5 - 48.1) | <0.001 |
| Control 5 | 72.8 (23.5) | 64.0 (61.4 - 75.4) |  | 56.5 (20.1 - 92.8) | 0.014 |
| Control 6 | 77.2 (18.1) | 70.8 (67.8 - 80.2) |  | 60.9 (37.6 - 84.1) | 0.001 |
| Control 7 | 80.0 (13.9) | 75.4 (70.5 - 85.0) |  | 63.7 (45.3 - 82.1) | <0.001 |
| Study hospital | 16.3 (5.8) | 14.1 (13.0 - 17.4) |  |  |  |
| Control group | 74.4 (19.7) | 70.1 (60.2 - 86.9) |  | 58.1 (37.7 - 78.6) | <0.001 |

SD, Standard Deviation; IQR, Interquartile range; CI, Confidence interval.
